# Common maternal and fetal genetic variants show expected polygenic effects on risk of small- or large-for-gestational-age (SGA or LGA), except in the smallest 3% of babies

**DOI:** 10.1371/journal.pgen.1009191

**Published:** 2020-12-07

**Authors:** Robin N. Beaumont, Sarah J. Kotecha, Andrew R. Wood, Bridget A. Knight, Sylvain Sebert, Mark I. McCarthy, Andrew T. Hattersley, Marjo-Riitta Järvelin, Nicholas J. Timpson, Rachel M. Freathy, Sailesh Kotecha

**Affiliations:** 1 Institute of Biomedical and Clinical Science, College of Medicine and Health, University of Exeter, Exeter, United Kingdom; 2 Department of Child Health, School of Medicine, Cardiff University, Cardiff, United Kingdom; 3 Center for Life Course Health Research, Faculty of Medicine, University of Oulu, Oulun yliopisto, Finland; 4 Unit of Primary Health Care, Oulu University Hospital, OYS, Oulu, Finland; 5 Wellcome Trust Centre for Human Genetics, University of Oxford, Oxford, United Kingdom; 6 Oxford Centre for Diabetes, Endocrinology and Metabolism, University of Oxford, Oxford, United Kingdom; 7 Oxford National Institute for Health Research (NIHR) Biomedical Research Centre, Churchill Hospital, Oxford, United Kingdom; 8 Department of Epidemiology and Biostatistics, MRC-PHE Centre for Environment and Health, School of Public Health, Imperial College London, London, United Kingdom; 9 Department of Life Sciences, College of Health and Life Sciences, Brunel University London, Kingston Lane, Uxbridge, Middlesex, United Kingdom; 10 Medical Research Council Integrative Epidemiology Unit, University of Bristol, Bristol, United Kingdom; Harvard School of Public Health, UNITED STATES

## Abstract

Babies born clinically Small- or Large-for-Gestational-Age (SGA or LGA; sex- and gestational age-adjusted birth weight (BW) <10^th^ or >90^th^ percentile, respectively), are at higher risks of complications. SGA and LGA include babies who have experienced environment-related growth-restriction or overgrowth, respectively, and babies who are heritably small or large. However, the relative proportions within each group are unclear. We assessed the extent to which common genetic variants underlying variation in birth weight influence the probability of being SGA or LGA. We calculated independent fetal and maternal genetic scores (GS) for BW in 11,951 babies and 5,182 mothers. These scores capture the direct fetal and indirect maternal (via intrauterine environment) genetic contributions to BW, respectively. We also calculated maternal fasting glucose (FG) and systolic blood pressure (SBP) GS. We tested associations between each GS and probability of SGA or LGA. For the BW GS, we used simulations to assess evidence of deviation from an expected polygenic model.

Higher BW GS were strongly associated with lower odds of SGA and higher odds of LGA (OR_fetal_ = 0.75 (0.71,0.80) and 1.32 (1.26,1.39); OR_maternal_ = 0.81 (0.75,0.88) and 1.17 (1.09,1.25), respectively per 1 decile higher GS). We found evidence that the smallest 3% of babies had a higher BW GS, on average, than expected from their observed birth weight (assuming an additive polygenic model: P_fetal_ = 0.014, P_maternal_ = 0.062). Higher maternal SBP GS was associated with higher odds of SGA P = 0.005.

We conclude that common genetic variants contribute to risk of SGA and LGA, but that additional factors become more important for risk of SGA in the smallest 3% of babies.

## Author summary

Babies in the lowest or highest 10% of the population distribution of birth weight (BW) for a given gestational age are referred to as Small- or Large-for-Gestational-Age (SGA or LGA) respectively. These babies have higher risks of complications compared with babies with BW closer to the average. SGA and LGA babies may have experienced growth restriction or overgrowth, respectively, but may alternatively just be at the tail ends of the normal growth distribution. The relative proportions of normal vs. sub-optimal growth within these groups is unclear. To examine the role of common genetic variation in SGA and LGA, we tested their associations with a fetal genetic score (GS) for BW in 11,951 European-ancestry individuals. We also tested associations with maternal GS (5,182 mothers) for offspring BW, fasting glucose and systolic blood pressure, each of which influences fetal growth via the *in utero* environment. We found fetal and maternal GS were associated with SGA and LGA, supporting strong maternal and fetal genetic contributions to birth weight in both tails of the distribution. However, within the smallest 3% of babies, the maternal and fetal GS for BW were higher than expected, suggesting factors additional to common genetic variation are more important in determining birth weight in these very small babies.

## Introduction

Size at birth is an important factor in new-born and infant survival. Term-born babies are most frequently admitted to the neonatal unit when born at the extremes of the birth weight distribution [1]. Small for Gestational Age (SGA; defined as birth weight adjusted for sex and gestational age that is below the 10^th^ percentile of the population or customized standard) is often used as a proxy indicator of fetal or intrauterine growth restriction (FGR or IUGR [2]). A fetus is described as growth-restricted when it has failed to reach its growth potential due to impaired placental function [3] or due to fetal or maternal reasons, and SGA fetuses are at higher risk of adverse outcomes such as stillbirth [4]. It is likely that SGA infants who are genetically small are at a lower risk of future morbidity than FGR infants. Risks of adverse outcomes are increased in preterm babies, and underlying mechanisms in preterm SGA babies are likely to be different to those born at term. SGA and FGR are often used interchangeably since fetal growth can be difficult to measure. [2,3] However, they are not synonymous: not all growth-restricted fetuses are small enough to be considered SGA [5], and the SGA group itself is heterogeneous with an estimated 50–70% being constitutionally small babies with normal placental function and outcomes [6], in addition to babies expected to be small due to chromosomal anomalies [7].

At the upper end of the birth weight distribution, large for gestational age (LGA, defined as sex- and gestational age-adjusted birth weight >90^th^ percentile) is associated with a higher risk of obstructed labour, which can lead to complications for both mother and baby, including injury, neonatal hypoglycaemia and even fetal death [8]. LGA may indicate excessive growth of the fetus, for example due to elevated maternal glycemia, which is a major determinant of fetal growth [9]. However, maternal fasting hyperglycemia only explains 2–13% of variation in birth weight [10,11], and most LGA babies are not born to mothers with diabetes [12]. This indicates that other factors are also important, for example, many LGA babies may be constitutionally large.

The contribution of common genetic variation to either SGA or LGA is not known, though associations between LGA and fetal genetic scores for birth weight have been demonstrated [13,14]. It is possible that a mismatch between a genetic score for birth weight and the actual, measured birth weight could help to identify babies who have either fallen short of, or exceeded, their growth potential. In clinical practice, this could be helpful if it improved the identification of FGR babies among those classified as SGA. Genetic studies of adult height previously investigated a similar question and showed that a genetic score composed of common variants was associated with adult height at the extremes of the population distribution [15]. However, the genetic score was not as extreme as expected in people with very short stature, suggesting that additional factors (e.g. rare mutations) were more important than common genetic variation in determining height in that group.

**Fig 1:**
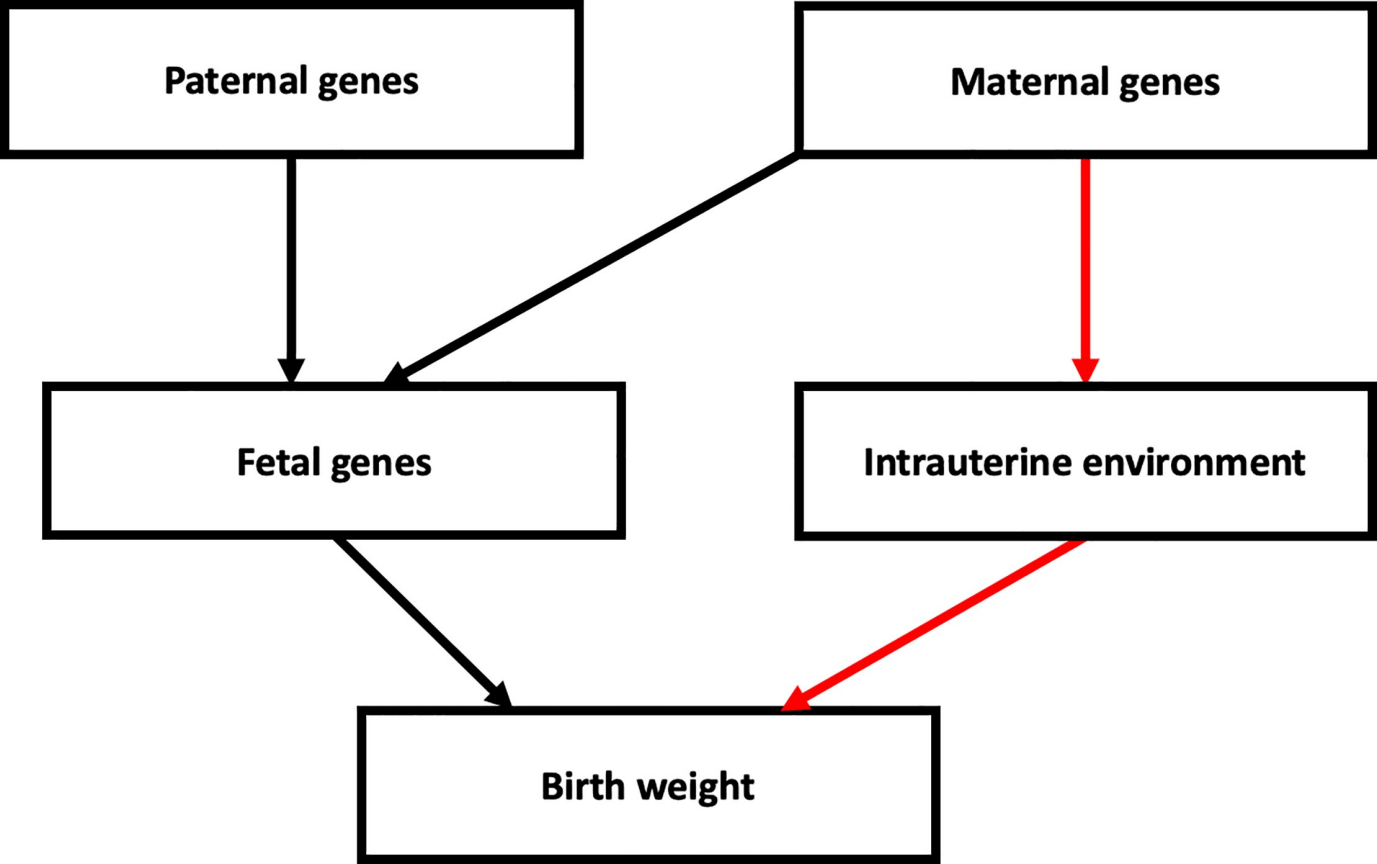
Diagram showing the possible pathways through which parental genotypes can influence birth weight. The black path represents direct fetal genetic effects on birth weight, and the red path represents maternal genetic factors which have an indirect effect on birth weight by modifying the intrauterine environment. This figure illustrates that, due to the correlation between maternal and fetal genotypes, genetic association analyses should model both maternal and fetal effects. Other environmental factors and gene-environment interactions that may influence birth weight are not shown.

In the current study, we investigated the genetic contribution to SGA and LGA in mothers and babies of European ancestry, using common genetic variants identified in the most recent genome wide association study (GWAS) of birth weight [16]. The genetic variants at the 190 most strongly-associated loci have individually small effects, but collectively explain 7% of the variation in birth weight. These genetic variants have either a direct fetal effect on birth weight (i.e. those inherited by the fetus and acting via fetal pathways), or an indirect maternal effect (i.e. through a primary effect on the intrauterine environment), or some combination of the two (Fig 1). The correlation between maternal and fetal genotypes means that associations between maternal genotype and birth weight can be confounded by fetal genotype effects, and vice-versa. To overcome this limitation, Warrington et al [16] estimated the independent maternal and fetal effect sizes at each of these loci. We used these independent effect sizes to calculate maternal and fetal genetic scores (GS) for birth weight. These GSs are designed to capture the independent maternal and fetal genetic contributions to birth weight. We tested for associations of these GSs with SGA and LGA. We then used simulations to test whether the GS in the SGA and LGA groups was consistent with an additive polygenic model in which there are many genetic variants, each contributing a small effect to the phenotype in an additive manner. Deviation from such a model might be observed if non-additive genetic effects, rare genetic variants with larger effects, or additional non-genetic factors were contributing to the risk of SGA or LGA. To investigate further the maternal genetic contribution to intrauterine factors known to be important across the birth weight distribution [16,17], we calculated genetic scores for fasting glucose (FG) and systolic blood pressure (SBP) and additionally tested their associations with SGA and LGA.

## Methods

### Cohort descriptions

Our analysis included a total of 6,769 term-born, singleton individuals with birth weight and fetal genotype data, from 2 studies, plus 5,182 mother-offspring pairs with maternal and fetal genotype data and birth weight from 2 further studies. Studies are described below, and summary data is shown in Table 1.

**Table 1:** Descriptive statistics of studies contributing to analysis. EFSOCH and ALSPAC sample size is the number of mother-child pairs in the analysis. NFBC1966 and NFBC1986 sample size is the number of offspring in the analysis.

Study	ALSPAC	EFSOCH	NFBC1966	NFBC1986
Country of origin	UK	UK	Finland	Finland
Year(s) of birth	1991–1993	2000–2004	1966	1985–6
Sample size (Male /Female (offspring sex))	4570 (2263/3207)	612 (320/292)	3691 (1839/1852)	3078 (1488/1590)
Data collection	Identified from obstetric data, records from the ALSPAC measurements, and birth notification	Measured within 12 hours of birth	Measured in hospitals	Measured in hospitals
Mean (SD) birth weight (grams) Males	3553 (491)	3585 (463)	3607 (506)	3626 (543)
Mean (SD) birth weight (grams) Females	3423 (450)	3447 (475)	3480 (466)	3519 (521)
Mean (SD) birth weight (grams)	3490 (476)	3519 (474)	3541 (489)	3572 (535)
Mean Maternal age (SD)	28.0 (4.96)	30.4 (5.28)	27.9 (6.5)	28.0 (5.3)
Mean Maternal Prepregnancy BMI (SD)	22.9 (3.83)	24.0 (4.45)	23.16 (3.18)	22.33 (3.38)
Median (IQR) GA (weeks) at delivery	40 (40–41)	40 (39–41)	40 (39–41)	40 (39–40)
Prevalence SGA	7.48% (b)	5.88% (b)	9.90% (c)	5.80% (c)
Prevalence LGA	10.01% (b)	11.60% (b)	11.80% (c)	15.50% (c)
TDI	Not available	0.25 (3.27)	Not available	Not available
Standard Occupational Class (a)	I 5.9% II 31.5% III (non-manual) 42.8% III (manual) 7.8% IV 9.9% V 2.1%	Not available	I 12.00% II 21.70% III 40.12% IV 9.78% Farmer I 15.04% Farmer II 1.35%	Professional/entrepreneur 18.8% Skilled, non-manual 17.6% Skilled, manual 41.0% Unskilled/apprentice 4.8% Farmer 7.9% Student/at home 4.0% Sick pension/unemployed 5.9%
% First Births	44.90%	49.3%	30.81%	34.0%
Smokers	24.60%	13.0%	19.08%	18.5%
Mean Maternal Height cm (SD)	164.0 (6.7)	165.0 (6.3)	160.0 (5.3)	163.0 (5.4)
Study description paper (PMID)	22507742; 22507743	16466435	19060910	31321419

(a) Derived from Office of Population Censuses & Surveys Standard Occupational Classification (Office of Population Censuses and Surveys (1991) Standard Occupational Classification. Her Majesty's Stationery Office)

(b) SGA and LGA defined using UK 1990 growth standards [23].

(c) SGA and LGA defined using the Swedish 1991 standards [24].

#### ALSPAC

The ALSPAC (Avon Longitudinal Study of Parents and Children) is a longitudinal cohort study covering the area of the former county of Avon, UK [18,19]. Women who were pregnant, living in the study area and had an expected delivery date between 1 April 1991 and 31 December 1992 were eligible for inclusion in the study. Birth weight and related data were abstracted from medical records. Children were genotyped on the Illumina HumanHap550 chip and mothers were genotyped using the Illumina human660W quad chip. Quality Control (QC) was undertaken as described previously [16] and imputation was to the HRC reference panel yielding data available for 8884 mothers and 8860 children with genotype data available. Of these, 4570 mother-child pairs with phenotype data were available for analysis.

#### EFSOCH

The Exeter Family Study of Childhood Health (EFSOCH) is a prospective study of children born between 2000 and 2004 in the Exeter region, UK, and their parents [20]. Birth weight measurements were performed as soon as possible after delivery. Individuals were genotyped using the Illumina Infinium HumanCoreExome-24 array. Genotype QC and imputation have been described previously [13]. After genotype QC, 938 mothers and 712 children with genotype data remained. Of these, 617 mother-child pairs with phenotype data were available for analysis.

#### NFBC1966

The Northern Finland Birth Cohort 1966 (NFBC1966) consists of mothers expected to give birth in the provinces of Oulu and Lapland in 1966 [21]. Birth weight and related characteristics were measured by examinations occurring directly after birth. Samples were genotyped on the Infinium 370cnvDuo. QC and imputation to the 1000 genomes reference panel were done centrally, resulting in 5400 children with genotype data. A total of 3691 children with phenotype data were available for analysis.

#### NFBC1986

The Northern Finland Birth Cohort 1986 (NFBC1986) consists of individuals born in the provinces of Oulu and Lapland between 1^st^ July 1985 and 30^th^ June 1986. Birth weight data was collected from hospital records after delivery. Following genotype QC, 3742 children with genotype data were available, with 3248 of these having phenotype data [22].

### Phenotype definitions

Since different mechanisms may lead to SGA and LGA in term and preterm infants, and in multiple births, we focused on term, singleton infants. Birth weight Z scores were calculated using growth standards separately for each cohort. The UK 1990 growth standards [23], were used for ALSPAC and EFSOCH and the Swedish 1991 standards [24] were use in NFBC 1966 and 1986 to adjust birth weight for sex and gestational duration. SGA was defined as birth weight z score < = -1.28, and LGA as birth weight z score > = 1.28. Controls were defined as those samples with birth weight z score > -1.28 for SGA and birth weight z score < 1.28 for LGA.

Since the use of growth standards results in different proportions of SGA and LGA by cohort, we conducted sensitivity analyses for comparison by defining SGA and LGA as the lower and upper 10% within each cohort. To do this, we regressed birth weight against sex and gestational age in term births (gestational age > = 37 weeks), and then calculated residuals from the regression model. Individuals with the smallest and largest 10% within each cohort of this residualised birth weight variable were defined as SGA and LGA respectively. Controls for comparison with SGA were taken as birth weight > = 10%, and for comparison with LGA as birth weight < = 90%. To test the effect of including babies classified as LGA in the control group for SGA and vice-versa, we conducted sensitivity analysis restricting the control group for both analyses to 10%< = birth weight< = 90%.

Genetic scores (GS) for birth weight, fasting glucose (FG) and systolic blood pressure (SBP) were calculated for all included individuals in each cohort, with higher GS corresponding to higher birth weight, FG or SBP, respectively. GS were calculated using Eq (1) where N_SNP_ is the total number of SNPs, w_i_ is the weight for SNP i and g_i_ is the genotype at SNP i. The same SNPs were included in the maternal and fetal GS. To calculate the SNP weightings, w_i_, since the cohorts used in our study were included in the largest available GWAS meta-analysis of birth-weight of Warrington et al [16], we re-ran the maternal and fetal GWAS meta-analyses of birth weight from that publication, but excluded the ALSPAC, EFSOCH, NFBC1966 and NFBC1986 studies. We used fixed effects meta-analysis implemented in Metal [25]. We then adjusted the effect estimates to estimate the independent maternal and fetal effects at each of the SNPs using the weighted linear model procedure of Warrington et al [16]. These adjusted effect estimates are designed to capture the independent maternal and fetal contribution at each SNP, which would otherwise be confounded by the correlation between maternal and fetal genotype. For the weights in the FG and SBP scores, we used effect estimates reported in large GWAS of FG and SBP, respectively, and the same weights were used for both maternal and fetal GS. SNPs and weights used in each score are given in S1–S3 Tables, along with details of the source publications.

$$GS=\frac{N_{SNP}}{\sum_{i}w_{i}}\sum_{i}w_{i}g_{i}$$ (1)

### Association analysis

#### Associations between SGA/LGA and maternal or fetal genetic scores for birth weight

Fetal genotypes (total n = 11,951) were available in ALSPAC (N = 4,570), EFSOCH (N = 612), NFBC1966 (N = 3,691) and NFBC1986 (N = 3,078). Associations were tested between the fetal GS for birth weight and outcomes (SGA/LGA) using logistic regression. Maternal genotypes were also available in ALSPAC (N = 4,570) and EFSOCH (N = 612). In these cohorts, associations between SGA/LGA and maternal GSs for birth weight were also tested in analogous regression models. Additional analyses including both maternal and fetal GS in the same regression model were performed to control for any residual correlation between maternal and fetal genotype. Results were meta-analysed using inverse variance weighted meta-analysis and heterogeneity between studies was assessed using Cochran’s Q test.

#### Investigating Deviations from the expected Polygenic Model in SGA/LGA

We performed simulations to assess whether there was any evidence of deviation from an expected additive polygenic model, under which we would not expect additional genetic variation or non-additive genetic effects in the tails of the birth weight distribution. Briefly, within ALSPAC and EFSOCH we calculated the associations of each fetal and maternal SNP with birth weight within each cohort, adjusted for maternal or fetal genotype, respectively. We simulated genotypes under the allele frequencies observed within each cohort. GSs were calculated for each simulated individual using simulated genotypes weighted by the within-cohort effect sizes. Phenotypes were then simulated as

$$p_{s}=x_{s}+{GS}_{s}$$

where p_s_ is the simulated phenotype for individual s, GS_s_ is the simulated genetic score for sample s and x_s_ is sampled from a normal distribution

$$x_{s}∼N\left(0,1-\sum_{i}\sigma_{{SNP}_{i}}^{2}\right)$$

Where $\sigma_{{SNP}_{i}}^{2}$ is the variance explained by SNP *i*. The mean GS within each bin of simulated phenotype was calculated. Simulations were performed 10,000 times to generate a simulated expected distribution of GS under an additive polygenic model. The observed mean GS was then compared to the expected distribution and an empirical p value was calculated. These p values were then meta-analysed.

Bins used in this analysis are 10% and 3% bins. Bin sizes of 3% were chosen because the rates of adverse outcomes were previously found to be highest in babies below the 3^rd^ centile [27].

#### Associations between SGA/LGA and maternal genetic scores for fasting glucose or systolic blood pressure

In ALSPAC and EFSOCH, where maternal genotypes were available, we tested the associations between the maternal GSs for FG and SBP, with outcomes (SGA/LGA) using linear regression, including sex and gestational age as covariates to control for residual confounding. We included both maternal and fetal GS in the same regression model to control for the correlation between maternal and fetal genotype. Results were meta-analysed using inverse variance weighted meta-analysis.

## Results

### Prevalence of SGA and LGA is strongly associated with maternal and fetal genetic scores for birth weight

**Fig 2:**
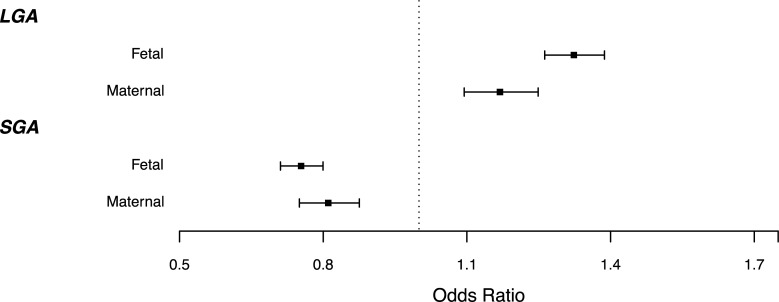
Odds of SGA or LGA per 1 decile higher fetal (N = 11,951; ALSPAC, EFSOCH, NFBC1966, NFBC1986) or maternal (N = 5,181; ALSPAC, EFSOCH) GS for birth weight. Error bars represent 95% confidence intervals, and weights used for fetal and maternal GS are independent of maternal and fetal effect respectively.

The minimum and maximum birth weights in our sample were 970g and 6080g respectively. The prevalence of SGA and LGA by percentile of fetal or maternal GS in ALSPAC (N = 4,570) is shown in S1 and S2 Figs, and the mean BW in ALSPAC by percentile of fetal and maternal GS is shown in S3 Fig. Both the fetal and maternal genetic scores for birth weight showed strong associations with LGA and SGA (Fig 2, S4A Table) in the expected directions. A one decile increase in the fetal GS for higher birth weight was associated with a greater odds of LGA (OR = 1.32 [95%CI: 1.26,1.39]; P = 7.0x10^-32^) and a lower odds of SGA (OR = 0.75 [0.71,0.80]; P = 8.5x10^-21^). Similarly, the maternal birth weight-raising GS showed strong associations with LGA (1.17 [1.09,1.25]; P = 3.2x10^-6^) and SGA (0.81 [0.75,0.88]; P = 7.7x10^-8^). Using Cochran’s Q to test heterogeneity between studies we found no strong evidence of heterogeneity among studies across all meta-analyses run other than fetal GS for birth weight (unadjusted for maternal genotype) and SGA (P = 0.008). These results were very similar to those of a sensitivity analysis of fetal GS conditional on maternal GS (and vice versa) in mother-child pairs (S4B Table), indicating that the use of the adjusted fetal and maternal weights [15] resulted in fetal and maternal GSs that were already independent of one another (S4 Table). Results were also consistent when using Appropriate for Gestational Age (AGA; 10th centile<BW<90th centile) as the control group, and when LGA and SGA were defined within each cohort as the largest and smallest 10% of babies, adjusted for sex and gestational age (S5A and S6A Table).

### Evidence that the GS in the lowest 3% of the population was higher than expected

**Fig 3:**
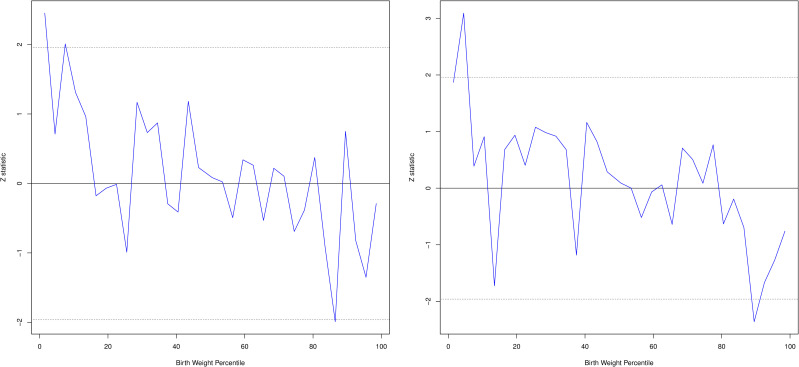
Difference between observed Z statistic for BW Z score (blue line) and simulated mean of birth weight GS (under a fully polygenic model; solid black line) and simulated upper and lower 95 percentiles (dotted black line) by 3% phenotype bins for fetal GS (left) and maternal GS (right) in ALSPAC.

Using simulation analyses in ALSPAC and EFSOCH (N = 5,182) we identified evidence of deviation from the expected additive polygenic model in the lowest 10% bin of the phenotype distribution (P_fetal_ = 0.001, P_maternal_ = 0.001). Repeating the analysis using 3% bins showed evidence of deviation in the lowest 3% bin for fetal birth weight GS (P = 0.0142). The maternal birth weight GS showed strong evidence of deviation in the 3–6% bin (P = 0.002), and weak evidence of deviation in the lowest 3% bin (P = 0.06; Fig 3; S7 Table). The maternal and fetal GS for birth weight were higher than expected given the birth weight within these groups, indicating that for a proportion of these babies their birth weight is lower than expected given their birth weight GS and assuming an additive genetic model. This finding indicates that factors other than common genetic variants, for example environmental or rare genetic factors, are acting to reduce birth weight for some individuals in this group.

There was no evidence of deviation from the expected additive polygenic model for the fetal GS in the top 10% of the phenotype distribution (P_fetal_ = 0.19). We saw some evidence of a lower maternal GS than expected in the top 10% group (P_maternal_ = 0.0074) but in the top 3% of the phenotype distribution there was no evidence of deviation (P_maternal_ = 0.44), but some evidence of a lower maternal GS than expected in the 88–91% group (P = 0.018; S7 Table).

### Prevalence of SGA and LGA are associated with maternal genetic scores for fasting glucose and SBP

**Fig 4:**
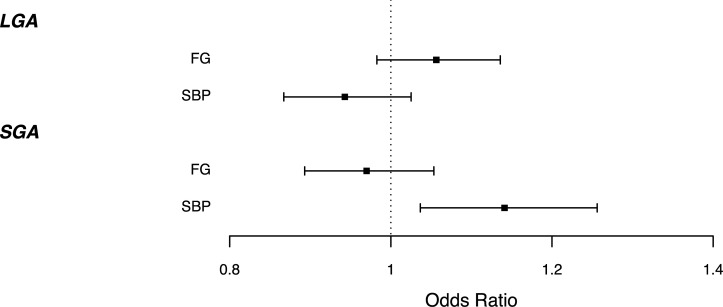
Odds of LGA/SGA 1 per decile higher maternal fasting glucose or SBP GS, corrected for fetal GS in ALSPAC and EFSOCH (N = 5,182). Error bars represent 95% confidence intervals.

The maternal GS for higher SBP was associated with higher odds of SGA (1.15 [1.04,1.27]; P = 4.7x10^-3^) (S8; S5B; S6B Table). This effect, observed across the genetic score distribution, is consistent with the known effects of maternal hypertension on birth weight. We found weak evidence that maternal GS for higher FG was associated with higher odds of LGA (1.06 [0.99,1.14]; P = 0.12) in accordance with the known effect of maternal hyperglycemia, although the 95%CI was wide and crossed the null. Warrington et al [16] previously demonstrated evidence of causal associations of both maternal FG and SBP with offspring BW across the term BW range. Since these associations are continuous across the birth weight distribution, our results show that lower maternal genetic susceptibility to raised SBP is associated with higher risks of SGA offspring. Associations of maternal SBP GS with LGA (0.93 [0.86–1.01]; P = 0.10) and FG GS with SGA (0.96 [0.89–1.05]; P = 0.39) were weak and the confidence intervals included the null (Fig 4, S8 Table). Sensitivity analysis defining SGA and LGA within each cohort showed evidence of association of maternal FG GS with LGA (S6A Table) suggesting that power to detect these associations is low in our sample and, given the known role of maternal hyperglycemia in LGA risk, which we would expect to be reflected in an association analysis of maternal FG GS and offspring birth weight, it would be informative to look at these associations in larger sample sizes.

## Discussion

We have shown that common birth weight-associated genetic variation in both the mother and the fetus contribute to the probability that term infants will be born small or large for gestational age. While these results indicate that a large proportion of those infants classified as SGA/LGA represent the tail ends of the normal distribution of birth weight, we also found some evidence of an excess of individuals with a higher GS for increased birth weight than expected under an additive polygenic model in the bottom 3% of the birth weight distribution.

The terms IUGR and SGA are often used interchangeably [26], which can imply that the majority of SGA babies have experienced restriction to their intrauterine growth, although it has been estimated [6] that 50–70% of babies classified as SGA are constitutionally small. There is a strong association in our study between fetal GS for higher birth weight and lower SGA risk. Together with the observed deviation from the polygenic model in only the smallest 3% of babies, our results suggest that the majority of babies in the SGA category are constitutionally small, and not growth-restricted. Our results are also consistent with the observation that the risk of adverse outcomes increases with decreasing birth weight within the SGA group, and the highest rates of adverse outcomes are in those with birth weight below the 3^rd^ centile [27].

We observed a higher GS than expected in the bottom 3% of birth weights, indicating that this group contains a proportion of babies whose birth weights are smaller than would be expected given their genetics. The demographics of the bottom 3% of individuals are presented in S9 Table. This group may be enriched for babies whose growth was inhibited by in-utero environmental factors, such as smoking. However, is it also possible that the presence of rare mutations with large effects on fetal growth within this group, which are not captured by the GS, contributed to the observed deviation. This finding would benefit from replication in larger studies to determine whether the deviation is limited to the smallest 3% of babies.

We found no evidence that the fetal GS for birth weight deviated from the polygenic model in the top 10% of the birth weight distribution, but there was some evidence of deviation for the maternal GS in this group. When we looked in smaller 3% bins, however, there was no evidence of association in the top 3% of the distribution. Overall our results do not suggest strong deviation from the polygenic model in babies with high birth weights, but we cannot rule out that smaller deviations would be detectable in a larger sample.

Although we have used a population based definition of SGA, customised birth weight standards including maternal height, weight and ethnic group, for defining SGA have been shown to increase association between SGA classification and neonatal morbidity and perinatal death compared to using population based definitions [2], although it has been suggested that this could be an artefact due to inclusion of more preterm births classified as SGA under this definition [28]. Babies born preterm have increased risk of neonatal morbidity and mortality compared to term babies, and it is likely that different mechanisms affect growth in preterm babies compared to IUGR babies born at term. Additionally, individuals classified as SGA by population-based growth standards but not by customised standards are not at increased risk of perinatal mortality and morbidity compared to those of appropriate weight [29–31]. Including genetics in the definitions of SGA has the potential to further refine the identification of babies who have failed to properly reach their growth potential, as shown by the association between SGA and birth weight GS demonstrated here.

Fetal genetic effects on birth weight represent the constitutional growth potential of the fetus, while maternal genetics influence birth weight indirectly by modifying the intrauterine environment [16]. The strong effects of the maternal GS for birth weight observed in our study indicate that maternal genetic variation acting via the *in utero* environment contributes to variation in SGA and LGA risk independently of the fetal genotype. Previous studies have shown that maternal FG and SBP have causal effects on birth weight in the normal range [15,16]. In women at highest genetic risk for raised FG and SBP, these could potentially contribute to LGA and SGA, respectively. We therefore investigated the associations between maternal genetic scores for FG or SBP and the risk of SGA or LGA. In line with known consequences of maternal gestational hypertension, a higher maternal SBP GS increased odds of SGA. The association of a 1 decile higher maternal SBP GS (OR = 1.15) is substantial in comparison to the effect of maternal hypertension on odds of SGA (OR = 1.35 [32]). A higher maternal FG GS showed weak evidence of association with higher odds of LGA in accordance with the known effects of maternal gestational diabetes, although the confidence intervals were wide. Given the known association between maternal gestational diabetes and LGA, replication in larger studies will be necessary to determine the potential contribution of lower maternal fasting glucose to SGA risk.

The current study has a number of strengths and limitations. Strengths include the fact that we have been able to construct independent maternal and fetal GS for birth weight. Separating maternal and fetal genetic contributions to birth weight is important because maternal and fetal genotype are correlated. This means that to avoid confounding, it is necessary to account for this correlation and obtain independent estimates of maternal and fetal genetic effects. To construct the GSs we chose to use only SNPs associated with the exposure (birth weight, SBP and FG) at a genome-wide threshold in their discovery samples. This decision ensures that the SNPs are robustly associated with the exposure and unlikely to also capture pleiotropic associations with other correlated phenotypes. In addition, the relationship between maternal and fetal genotype causes difficulty in estimating independent maternal and fetal genetic associations at each SNP. Limiting the SNPs to those with good power to estimate these independent associations improves the separation of maternal and fetal effects of the GSs. Both a limitation and strength of our study is that all of the cohorts used were of European ancestry. The GSs used in this study were discovered in studies of European ancestry and generalisability to studies of non-European ancestry has not been widely tested. In addition, well powered cohorts of mother-child pairs with genotype data as well as birth weight data are not currently available, meaning that the conclusions of the present study are not necessarily generalisable to individuals of non-European ancestry. As such there is a need for genetic studies in populations of ancestries other than Europeans. The use of only singleton, term babies in our analysis means that our results do not necessarily translate to pre-term babies or multiple births. While we had sufficient sample size to estimate the association of birth weight GS with SGA/LGA, the limited number of mother-child pairs which were available to examine the associations of FG and SBP GS with SGA/LGA meant that these estimates had large confidence intervals. Larger numbers of mother-child pairs would allow for more precise estimates, for example of the association of low SBP on LGA which has potentially clinically relevant implications. Although SGA and FGR are often used synonymously, there are differences between the terms. In our study do not have information required to distinguish FGR babies from SGA ones, meaning that we were not able to examine the association between BW GS and FGR specifically.

Our analysis has shown that common birth weight-associated genetic variation contributes to the risk of babies being born small or large for gestational age. We found evidence of deviation from the polygenic model in the smallest 3% of babies, consistent with enrichment for fetal growth restriction in this group.

## Supporting information

S1 Fig Fraction of babies born SGA (left) or LGA (right) by percentile bins of fetal birth weight GS in ALSPAC (N = 4,569).
(PNG)

S2 Fig Fraction of babies born SGA (left) or LGA (right) by percentile bins of maternal birth weight GS in ALSPAC (N = 4,569).
(PNG)

S3 Fig Mean birth weight in ALSPAC by percentile bins of fetal (left) and maternal (right) birth weight GS (N = 4,569).
(PNG)

S1 Table SNPs and weights used to construct fetal and maternal birth weight GS. Source PMID:31043758.
(XLSX)

S2 Table SNPs and weights used to construct fasting glucose GS. (XLSX)

S3 Table SNPs and weights used to construct SBP GS. (XLSX)

S4 Table A) Associations between fetal and maternal birth weight GS and LGA/SGA. Fetal GS associations include ALSPAC, EFSOCH, NFBC86 and NFBC66. Maternal GS associations include ALSPAC and EFSOCH. Odds ratios represent the increase in risk per decile of birth weight GS. B) Associations between fetal and maternal BW GS and LGA/SGA, adjusted for maternal and fetal BW GS respectively in ALSPAC and EFSOCH mother-child pairs.
(XLSX)

S5 Table A) Associations between Fetal and Maternal BW GS and LGA/SGA. The control group consists of babies with birth weight >10th centile and <90th centile. Odds ratios represent the increase in risk per decile of BW GS. B) Associations between Maternal FG and SBP GS, adjusted for fetal genotype, and LGA/SGA. The control group consists of babies with birth weight >10th centile and <90th centile. Odds ratios represent the increase in risk per decile of FG/SBP GS.
(XLSX)

S6 Table A) Associations between Fetal and Maternal BW GS and LGA/SGA defined within each cohort. Odds ratios represent the increase in risk per decile of BW GS. B) Associations between Maternal FG and SBP GS, adjusted for fetal genotype, and LGA/SGA defined within each cohort. Odds ratios represent the increase in risk per decile of FG/SBP GS.
(XLSX)

S7 Table Results from meta-analysis of simulations in ALSPAC and EFSOCH to analyse deviations from the expected polygenic model. (XLSX)

S8 Table Associations between Maternal FG and SBP GS and LGA/SGA, with adjustment for fetal GS un ALSPAC and EFSOCH mother-child pairs. Associations include mother-child pairs from ALSPAC and EFSOCH. Odds ratios represent the increase in risk per decile of FG/SBP GS.
(XLSX)

S9 Table Descriptive statistics of the lowest 3% of babies in EFSOCH and ALSPAC. (XLSX)
