# Supplementary figures and images for: Common maternal and fetal genetic variants show expected polygenic effects on risk of small- or large-for-gestational-age (SGA or LGA), except in the smallest 3% of babies
Source: PLoS Genet. 2020 Dec 7;16(12):e1009191. doi: 10.1371/journal.pgen.1009191 (PMC7721187; doi:10.1371/journal.pgen.1009191)

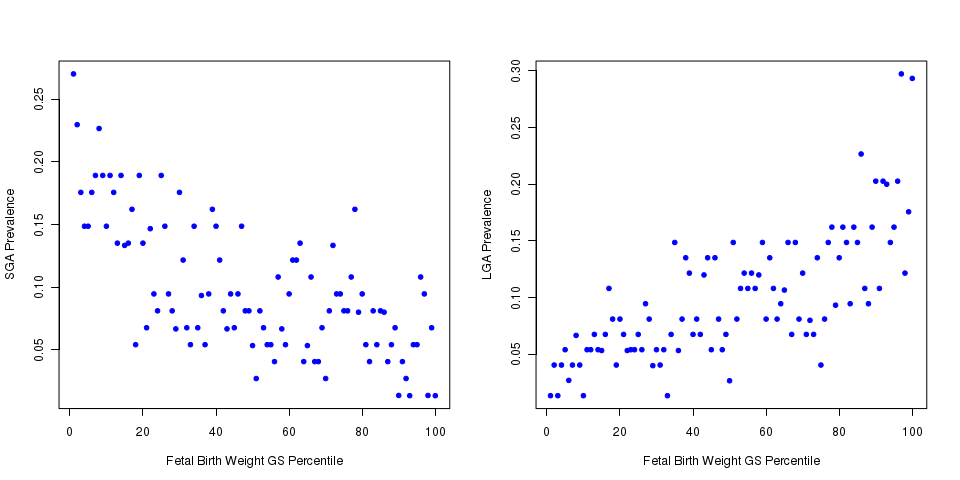

Supplement: S1 Fig — Fraction of babies born SGA (left) or LGA (right) by percentile bins of fetal birth weight GS in ALSPAC (N = 4,569). (PNG) [file pgen.1009191.s001.png]

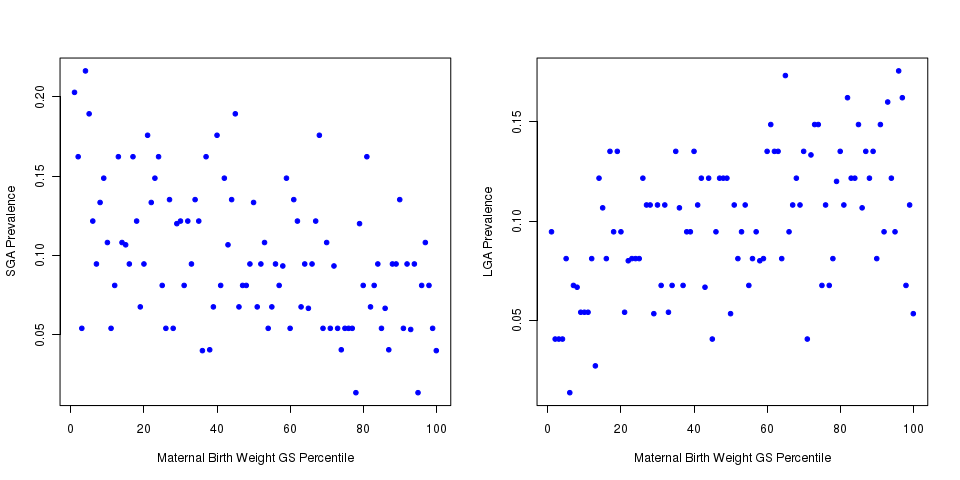

Supplement: S2 Fig — Fraction of babies born SGA (left) or LGA (right) by percentile bins of maternal birth weight GS in ALSPAC (N = 4,569). (PNG) [file pgen.1009191.s002.png]

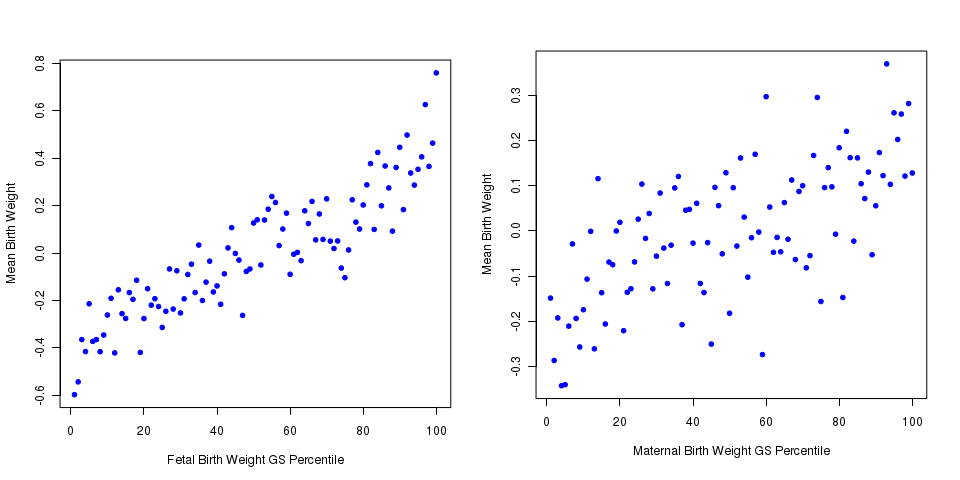

Supplement: S3 Fig — Mean birth weight in ALSPAC by percentile bins of fetal (left) and maternal (right) birth weight GS (N = 4,569). (PNG) [file pgen.1009191.s003.png]
